# A Quantitative and Radiomics approach to monitoring ARDS in COVID-19 patients based on chest CT: a retrospective cohort study

**DOI:** 10.7150/ijms.48432

**Published:** 2020-07-06

**Authors:** Yuntian Chen, Yi Wang, Yuwei Zhang, Na Zhang, Shuang Zhao, Hanjiang Zeng, Wen Deng, Zixing Huang, Sanyuan Liu, Bin Song

**Affiliations:** 1 Department of Radiology, West China Hospital, Sichuan University, Chengdu 610041, China.; 2 Department of Research and Development, Shanghai United Imaging Intelligence Co., Ltd. Shanghai 200232, China.; 3 Department of Endocrinology, West China Hospital, Sichuan University, Chengdu 610041, China.; 4 Department of Radiology, Chengdu Public Health Clinical Medical Center, Chengdu 610066, China.

**Keywords:** COVID-19, Computed tomography, Acute respiratory distress syndrome, Radiomics, Quantitative

## Abstract

**Rationale:** Acute respiratory distress syndrome (ARDS) is one of the major reasons for ventilation and intubation management of COVID-19 patients but there is no noninvasive imaging monitoring protocol for ARDS. In this study, we aimed to develop a noninvasive ARDS monitoring protocol based on traditional quantitative and radiomics approaches from chest CT.

**Methods:** Patients diagnosed with COVID-19 from Jan 20, 2020 to Mar 31, 2020 were enrolled in this study. Quantitative and radiomics data were extracted from automatically segmented regions of interest (ROIs) of infection regions in the lungs. ARDS existence was measured by Pa02/Fi02 <300 in artery blood samples. Three different models were constructed by using the traditional quantitative imaging metrics, radiomics features and their combinations, respectively. Receiver operating characteristic (ROC) curve analysis was used to assess the effectiveness of the models. Decision curve analysis (DCA) was used to test the clinical value of the proposed model.

**Results:** The proposed models were constructed using 352 CT images from 86 patients. The median age was 49, and the male proportion was 61.9%. The training dataset and the validation dataset were generated by randomly sampling the patients with a 2:1 ratio. Chi-squared test showed that there was no significant difference in baseline of the enrolled patients between the training and validation datasets. The areas under the ROC curve (AUCs) of the traditional quantitative model, radiomics model and combined model in the validation dataset was 0.91, 0.91 and 0.94, respectively. Accordingly, the sensitivities were 0.55, 0.82 and 0.58, while the specificities were 0.97, 0.86 and 0.98. The DCA curve showed that when threshold probability for a doctor or patients is within a range of 0 to 0.83, the combined model adds more net benefit than “treat all” or “treat none” strategies, while the traditional quantitative model and radiomics model could add benefit in all threshold probability.

**Conclusions:** It is feasible to monitor ARDS from CT images using radiomics or traditional quantitative analysis in COVID-19. The radiomics model seems to be the most practical one for possible clinical use. Multi-center validation with a larger number of samples is recommended in the future.

## Introduction

The coronavirus disease 2019 (COVID-19) caused by novel coronavirus SARS-CoV-2 has been spreading rapidly in the world 1,2. Compared with the previous respiratory epidemics, there are some new characteristics of COVID-19 and new patient management challenges. For instance, studies indicated that COVID-19 patients could be asymptomatic and highly contagious in the early stage, resulting in difficulty for early diagnosis 3,4. Fortunately, since the disease outbreak, there a good few early diagnosis models on COVID-19 were published 5,6. Some studies indicated that Computed Tomography (CT) findings might be earlier than the symptom onset in COVID-19 patients 7, and hence CT imaging had become a major complementary tool for diagnosis and assessment of COVID-19 8. However, there were still a lot that radiologists could do in COVID-19 management, such as monitoring of the disease progression or prediction of the patients' prognosis.

One of the challenges in treatment of COVID-19 is how to decrease the mortality rate and improve treatment outcome. Acute respiratory distress syndrome (ARDS) is the major cause of severe cases, and early detection and early treatment of ARDS patients could improve the outcome 9,10.

However, it is a tough task for clinicians to be conscious of early ARDS existence in COVID-19 because there could be no symptom deterioration or abnormalities of laboratory tests before the mild ARDS existence 11. The most reliable way to overcome this difficulty is to perform arterial gas blood analysis frequently, which was the gold standard of the ARDS diagnosis, but the arterial puncture was an invasive procedure and could cause extra risk for complications.

ARDS is caused by the injury of alveolar-capillary membrane 12, which could result in imaging feature changes captured using quantitative analysis from chest CT images 13. Therefore, it is possible to use the traditional quantitative chest CT metrics, such as volume and density to monitor the existence of ARDS. However, to our knowledge, no studies have used quantitative results to monitor the ARDS in COVID-19, while few quantitative results were used in some diagnosis models 14.

It should be noted that computing the aforementioned quantitative changes is not trivial by traditional methods. Radiomics method thus is ideal to be used in this situation for extracting rich image features. Such kind of method refers to extracting a large number of imaging features in the high-content manner, and use high-dimensional feature selection and classification methodologies for analyzing the relationship between imaging features and clinical factors 15. Radiomics methods have been successfully applied in various applications including some infectious diseases 16-20. However, there is no radiomics-based study for early detection of ARDS in COVID-19 patients.

In this study, we use quantitative data analysis of chest CT images to detect the existence of ARDS during the COVID-19 treatment. The imaging data were analyzed by traditional and radiomics approaches, respectively, and their performances were validated and compared using the datasets collected from our hospital.

## Methods

### Patients' cohort and clinical data collection

All COVID-19 patients treated in Chengdu Public Health Center between Jan 20, 2020 and Mar 31, 2020 were enrolled in our study. The diagnosis of COVID-19 was based on a positive result high-throughput sequencing or real-time reverse-transcriptase-polymerase-chain-reaction (RT-PCR) assay of nasal and pharyngeal swab specimens 21. After collecting the CT imaging and clinical management data, a subset of patients were excluded according to the following criteria: (i) age < 18 years-old; (ii) incomplete medical records; (iii) cases with no arterial blood analysis result corresponding to respective CT images.

The research protocol was approved by the appropriate ethics review board of our hospital, and patient informed consent form was waived because only anonymized data were used, and no diagnosis and treatment for patients has been altered due to this retrospective study.

Clinical data, such as age, sex, arterial blood analysis results and the numbers of comorbid were obtained from the medical records. The comorbid diseases included: COPD, hypertension, hyperlipemia, cerebral infarction, coronary heart disease, cardiac dysfunction III-IV, Liver dysfunction, diabetes, chronic kidney disease and, malignant tumor. The ARDS existence was measured by the result of arterial blood analysis. If the Pa0_2_/Fi0_2_ of the artery was <300, the patient was considered as with ARDS.

### CT image acquisition and traditional quantitative metrics extraction

Non-contrast chest CT examinations were performed for each patient when their doctors deemed it was necessary to assess their respiratory status. CT examination was prohibited when the patient could not get rid of the ventilator. Details of CT scanning were provided in supplementary materials **(Table S1)**. Qualitative assessment was performed by two independent radiologists after each CT examination, including the change of volumes, density and location of lesions.

Infection regions were segmented by a pulmonary pneumonia-dedicated multi-task deep learning algorithm, trained by using over 6000 multi-center CT scans (United Imaging Intelligence) based on VB-Net 22. Its accuracy was tested by two expert radiologists with 15 years (Zixing Huang) and 25 years (Bin Song) experience in chest CT interpretation. More detailed information of segmentation algorithm was shown in the supplementary material **(Table S2)**.

The following traditional quantitative metrics were calculated to quantify infectious regions of the image of each patient:

- Volumes of infection in the whole lung, and volumes of infection in each lobe and each bronchopulmonary segment;
- Percentage of Infection (POI) in the whole lung, each lobe and each bronchopulmonary segment;
- Hounsfiled Unit (HU) histograms within different infection regions. Different HU ranges or components were used, including (zone 1: <-750), (zone 2: from -750 to -650), (zone 3: -649 to -550), (zone 4: -549 to -450), (zone 5: -449 to -350), (zone 6: -349 to -250), (zone 7: -249 to -150), (zone 8: -149 to -50), (zone 9: -49 to 100) and (zone10:>100) inside the infection region.

**Figure 1:**
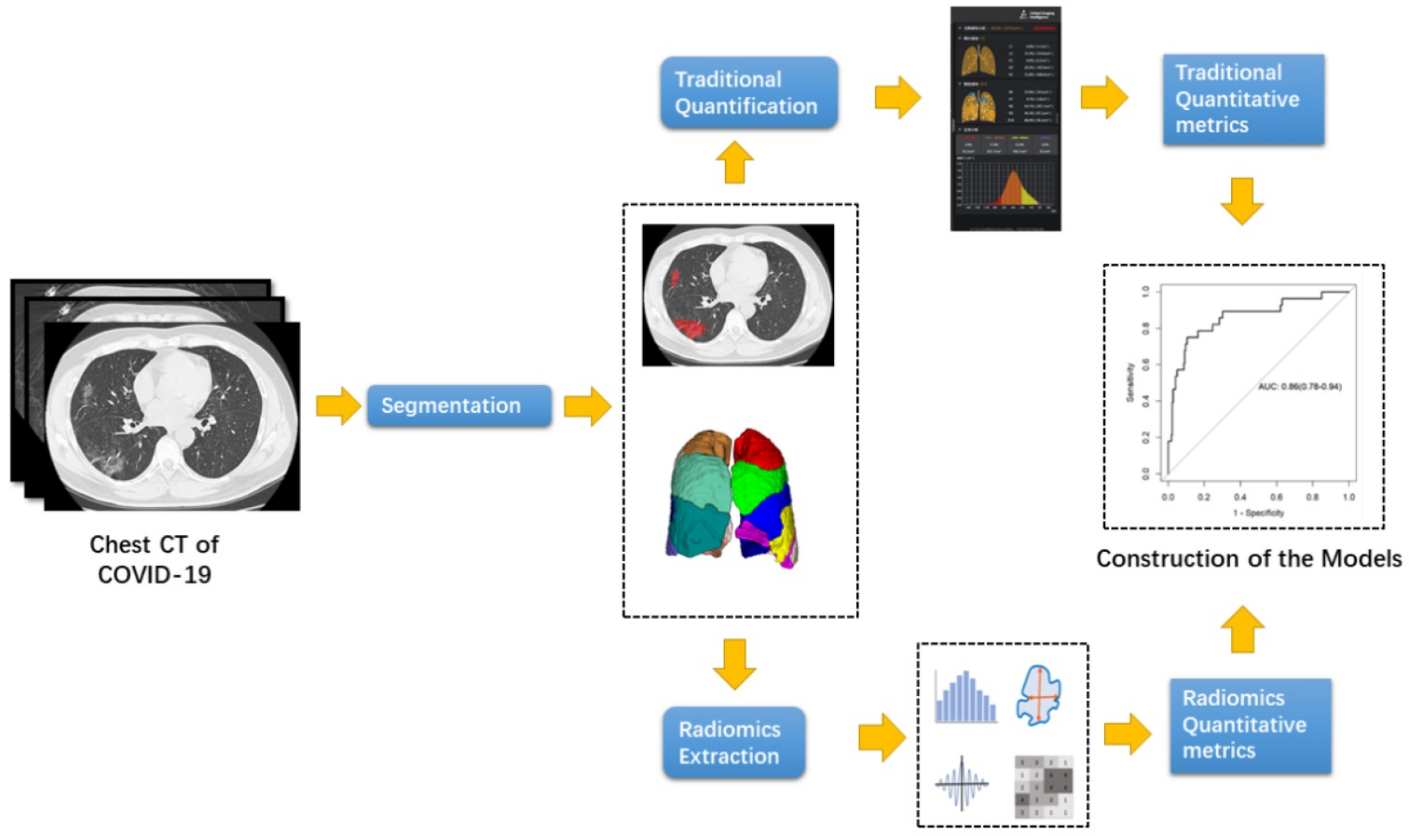
Pipeline of the traditional quantitative metrics and radiomics metric extraction.

The entire pipeline for the traditional quantitative COVID-19 extraction was shown in **Figure 1.**

A two-step logistic regression was performed to explore the relationship between traditional quantitative metrics and ARDS existence. First, a univariable logistic regression was performed on all clinical and quantitative imaging features. Then a multivariable logistic regression was performed on factors whose *P* value <0.1 in the first regression. Finally, a linear combination of the above significant factor was applied to build a traditional quantitation predictive model.

### Radiomic feature extraction

The radiomics workflow is presented in **Figure 1.** ROIs were the same regions used for traditional quantitative assessment, which were segmented automatically. Texture extraction was performed using Pyradiomics in Python 3.7. All radiomics features were based on Image Biomarkers Standardization Initiative (IBSI). In summary, 104 imaging features were extracted from individual CT, including 18 first-order features, 14 shape features, 16 glrlm features, 14 gldm feautures, 16 glszm features, 21 glcm features and 5 ngtdm features.

Radiomics features were all normalized by StandardScaler in both datasets. Then, a two-step high-dimensional data reduction was performed. First, minimum redundancy and maximum correlation of feature selection (mRMR) was performed to eliminate the redundant and irrelevant features, and 30 features were retained. Then, the least absolute shrinkage and selection operator (LASSO) logistic regression algorithm was applied to choose the optimized subset of features to construct the final model. A linear regression was performed by combination of selected features that were weighted by their respective LASSO results. A risk score, called radiomics score was calculated by the formula for each patient to refer the risk of ARDS existence. A radiomics model was constructed based on the radiomics score. Finally, a combination of quantitation and radiomics model was constructed based on the multivariable regression result of the selected quantitative variable and radiomics scores.

### Evaluation of the constructed model

The predictive performance of the constructed models was assessed by ROC, where AUC was calculated for the quantification in both training set and validation sets. Also, DCA was performed by calculating the net benefits for a range of threshold probabilities in the training and validation sets.

### Statistical analysis

Continuous variables were reported as the mean (standard deviation) or median (interquartile range [IQR]). Student's t-test or Mann-Whitney U test was used to compare between-group differences (presence and non-presence of primary composite endpoints) based on distributions. Categorical variables were presented as n (%) and compared using Chi-square (χ^2^) test or Fisher's exact test. The LASSO logistic regression model was performed with penalty parameter tuning, which was conducted by 10-fold cross-validation by minimum criteria. Back-ward step-down selection was applied to the multivariable model.

All statistical tests were performed using R statistical software version 3.6.3. “mRMRe” package was used for the mRMR reduction; “glmnet” package was used for the LASSO logistic regression; “pROC” package was used for ROC curves plotting; “dac.R” package was used for DAC analysis. Statistical significant was considered when a two-sided *P* <0.05.

## Results

### Patient characteristics

Totally, 102 COVID-19 patients were enrolled in our study. 4 patients were excluded due to age <18. 14 patients were excluded because of incomplete medical records. Finally, 84 patients were included in their study. There were 381 CT scans for these patients, and 352 CT scans had corresponding arterial blood gas analysis results.

The median age of patients was 49 (IQR: 34.00 - 60.75), male proportion was 61.90% (52/84). Among these patients, 61.90% (52/84) patients were mild, 32 patients were severe and 15.47% (13/84) patients were transferred into ICU. The median time from symptom onset to admission was 5 days (IQR: 3 - 9 days). The median number of CTs during the admission was 4 (3-5). The most common initial symptom was fever (80.95%, 68/84) and cough (70.23%, 59/84). The most common comorbidity was hypertension (20/84, 23.8%). The detail of patients characterize was shown in **Table 1.**

### Clinical data and traditional quantitative metrics

Among 352 CT scans, 14.49% (51/352) were shown to have ARDS existence at that moment. Qualitative assessment showed that 47.72% of CT results were better than the previous ones, while 26.70% CT images showed significant deterioration of infection.

Quantitative assessment of CT showed the mean infectious proportion of the lung was 6.38 ± 8.69%. The mean density of infection regions was -588.96 ± 134.34 HU. The largest component of the infection region was Zone 1 (HU < -750), which composed 12.38 ± 11.15% of infection region among all patients on average. The mean infection area proportion of inner zone of lung was 3.30% ± 5.35%, while the mean proportion of peripheral zone of lung was 3.07% ± 4.00%. More information on the area proportion of different density interval region could be achieved in** Table 2**.

Χ^2^ test was applied to all clinical data to assess the distribution of each factor in mild group and severe group. The factor with *P* value<0.1 was put into further logistic regression. The χ^2^ result showed that Male proportion (*P*=0.089), chronic kidney disease (*P*= 0.067), coronary heart disease (*P*=0.002), cardiac dysfunction (*P*=0.001), COPD (*P*=0.028), and hypertension (*P* = 0.01) met the above criteria.

**Figure 2:**
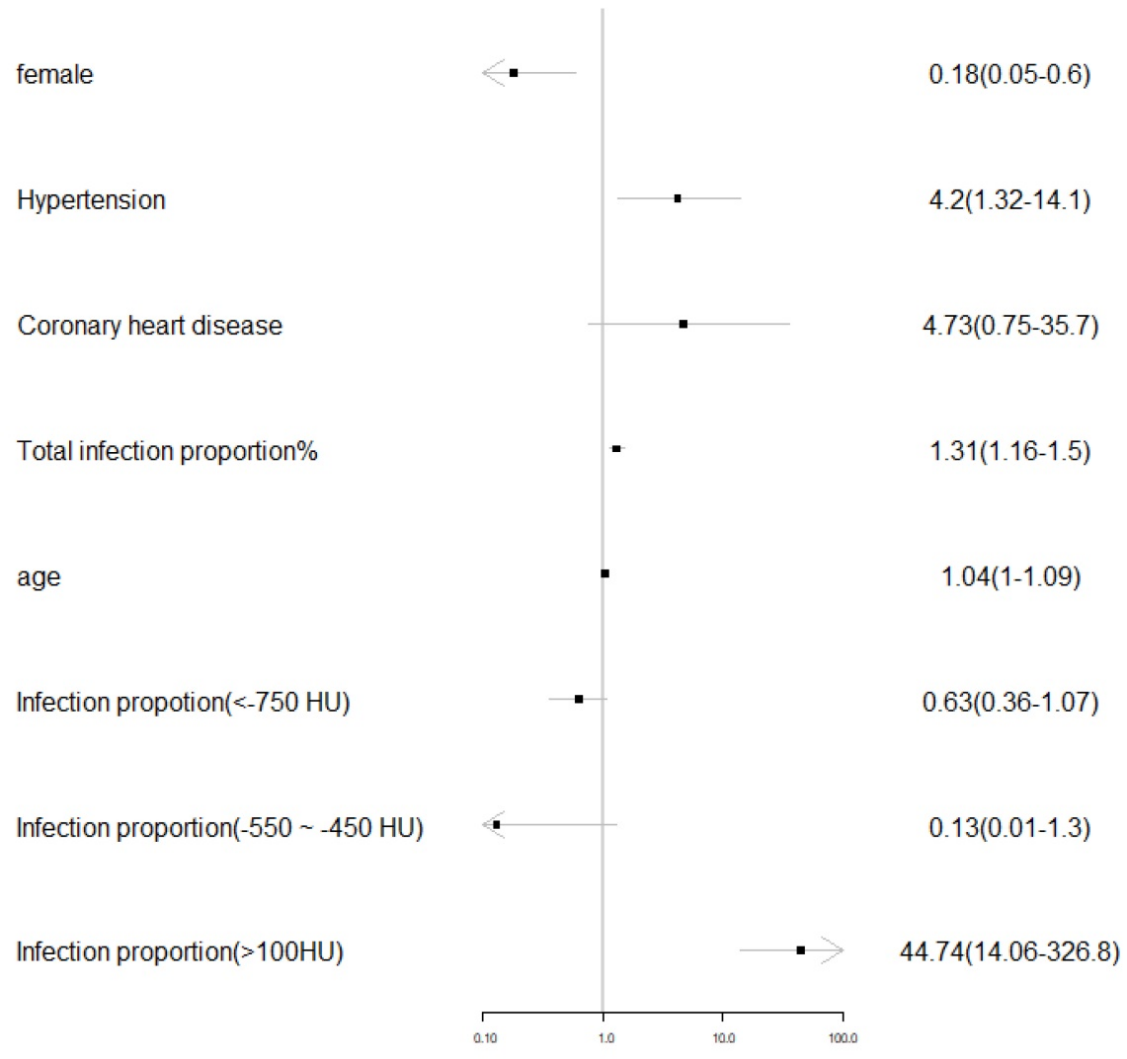
Visualization of the multivariable logistic regression results on the traditional quantitative and clinical metrics.

A multiple variable backward step logistic regression was applied to all traditional metrics with all the above including clinical variables. The regression result showed that male (*P*=0.008), existence of hypertension (*P*= 0.016), total infection proportion (*P*<0.001), age (*P*=0.048) and area proportion of zone 10 (CT value >100 HU) were significantly related to the ARDS existence. The regression result was visualized in **Figure 2.**

### Radiomics signature construction

**Figure 3:**
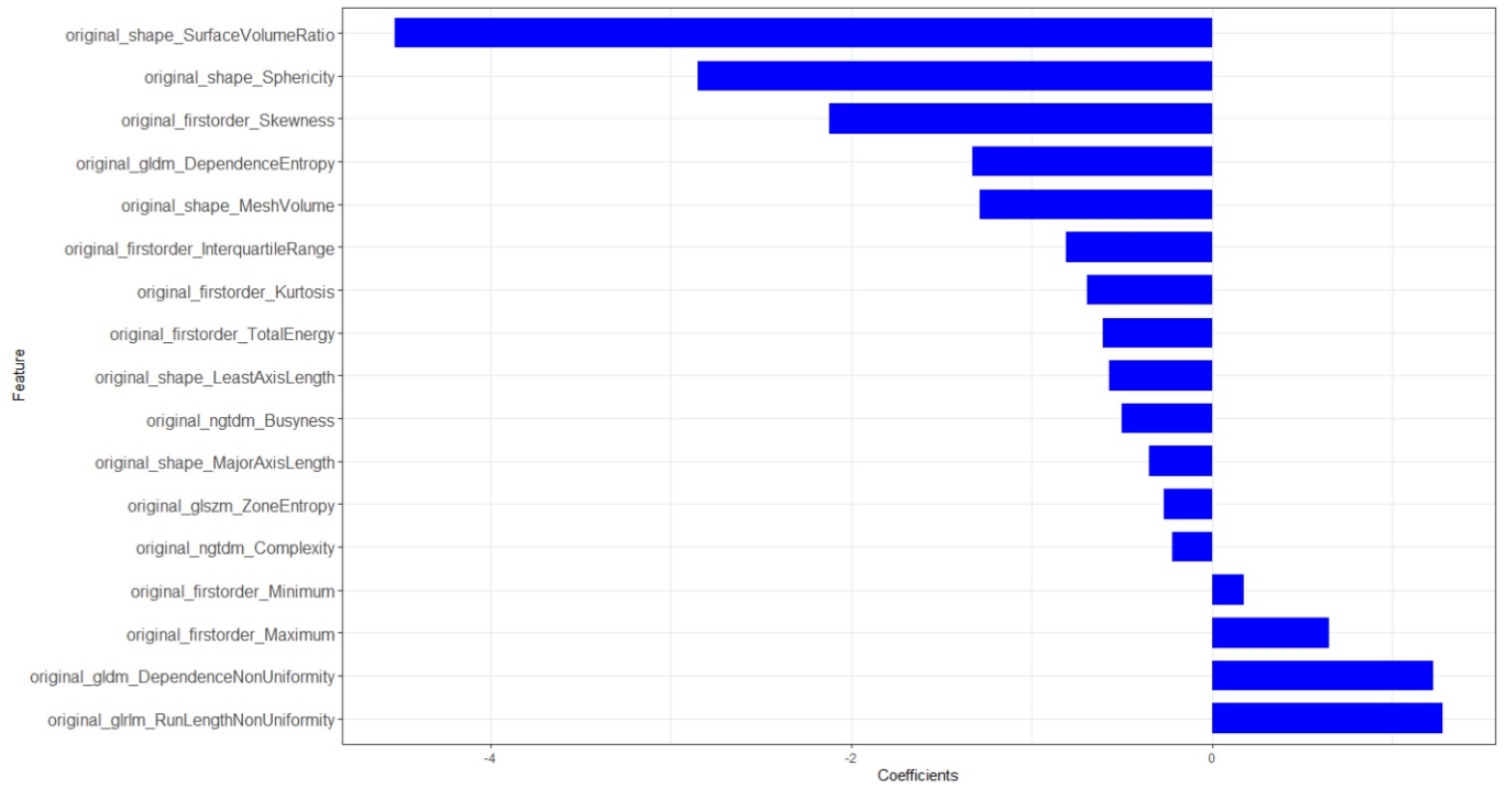
Histogram of selected radiomics features. X labels indicates the coefficients of each feature in the lasso regression.

**Figure 4:**
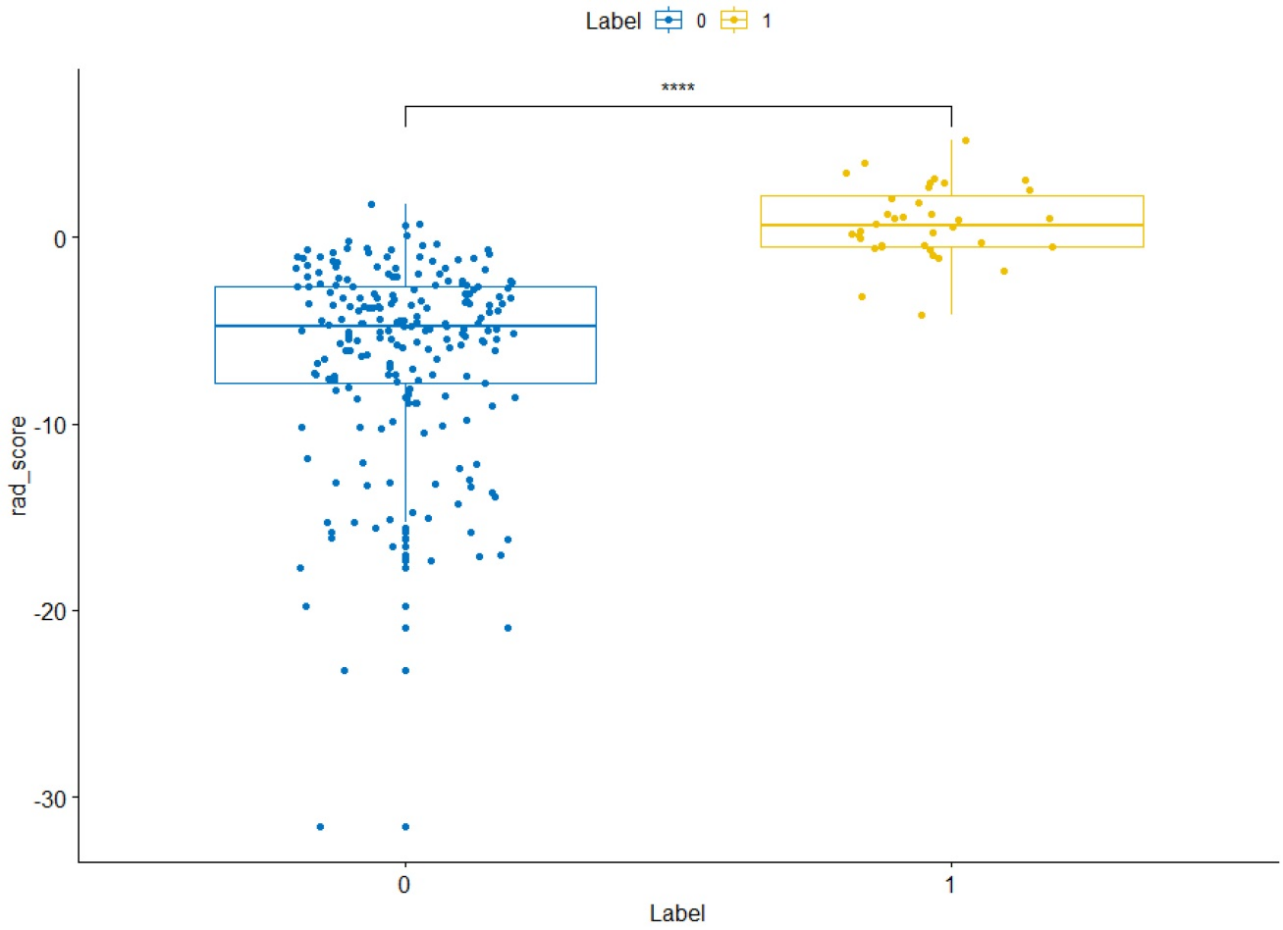
** Radiomics score distribution in the training sets.** “0” group represents the cases without ARDS existence. “1” group represents the cases with ARDS existence. NS, *,**,***,**** means the *P* value of wilcox test between two group >0.05, <0.05, <0.01, <0.001, <0.0001 respectively.

**Figure 5:**
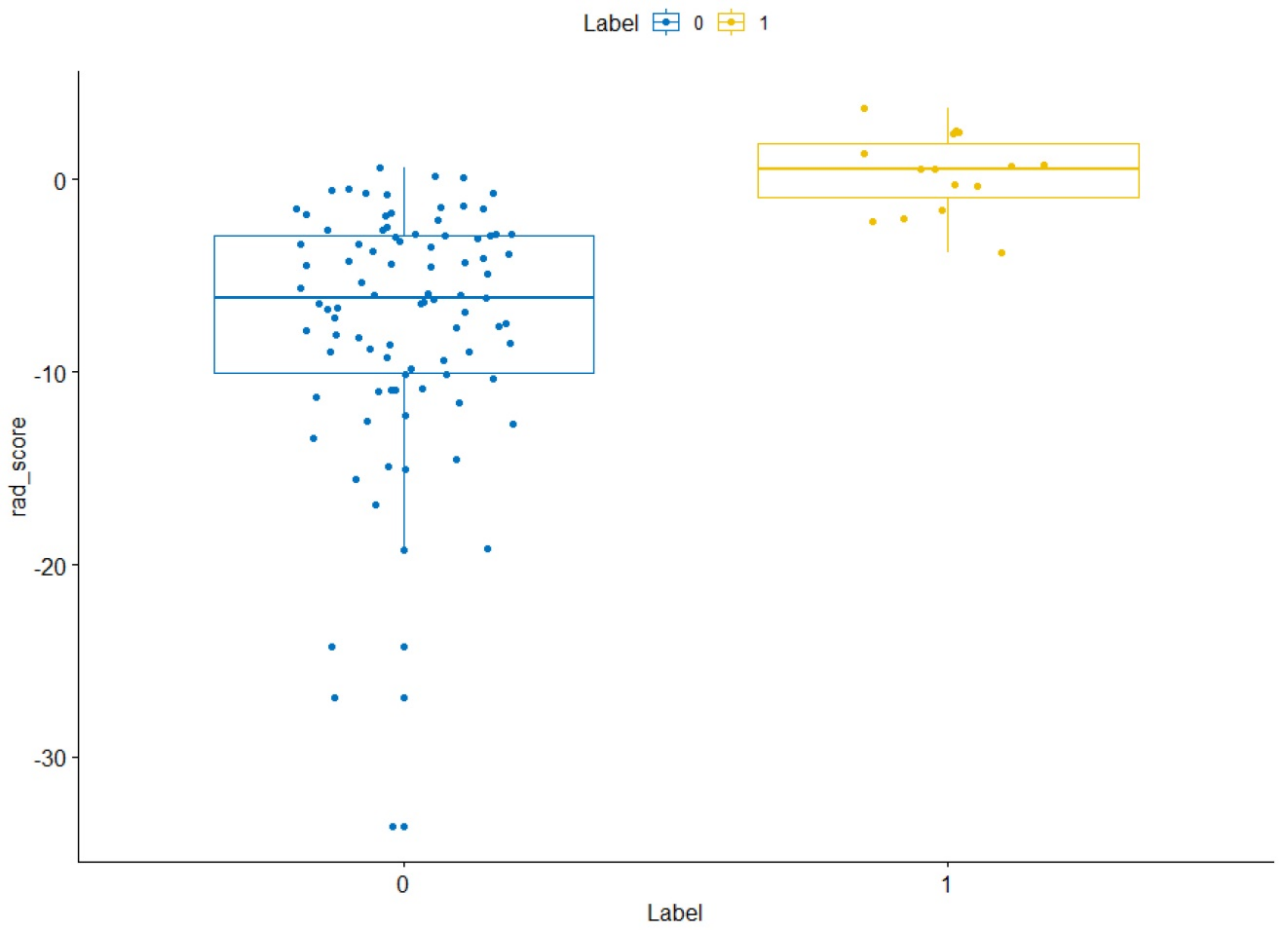
** Radiomics score distribution in the validation sets.** “0” group represents the cases without ARDS existence. “1” group represents the cases with ARDS existence. NS, *, **, ***, **** means the *P* value of wilcox test between two group >0.05, <0.05, <0.01, <0.001, <0.0001 respectively.

The training sets and validation sets were generated by random sampling from the CT image cohort with a ratio of 2:1.The χ^2^ test showed that there was no difference (χ2 < 0.001, *P*-value= 0.99) of the number of ARDS existence case in train sets (36/247) and validation sets (15/105). There was no difference between training sets and validation sets **(Table S3).** A total of 104 imaging features were extracted from each CT image. After mRMR reduction and lasso regression, 17 features were selected to calculate the radiomics score **(Figure 3).** The details of radiomics score calculation method were demonstrated in the supplementary material. The Wilcoxon test showed that the distribution of radiomics score was significantly different in both training sets and validation sets **(Figures 4 & 5)**.

### Model combination and assessment

**Figure 6:**
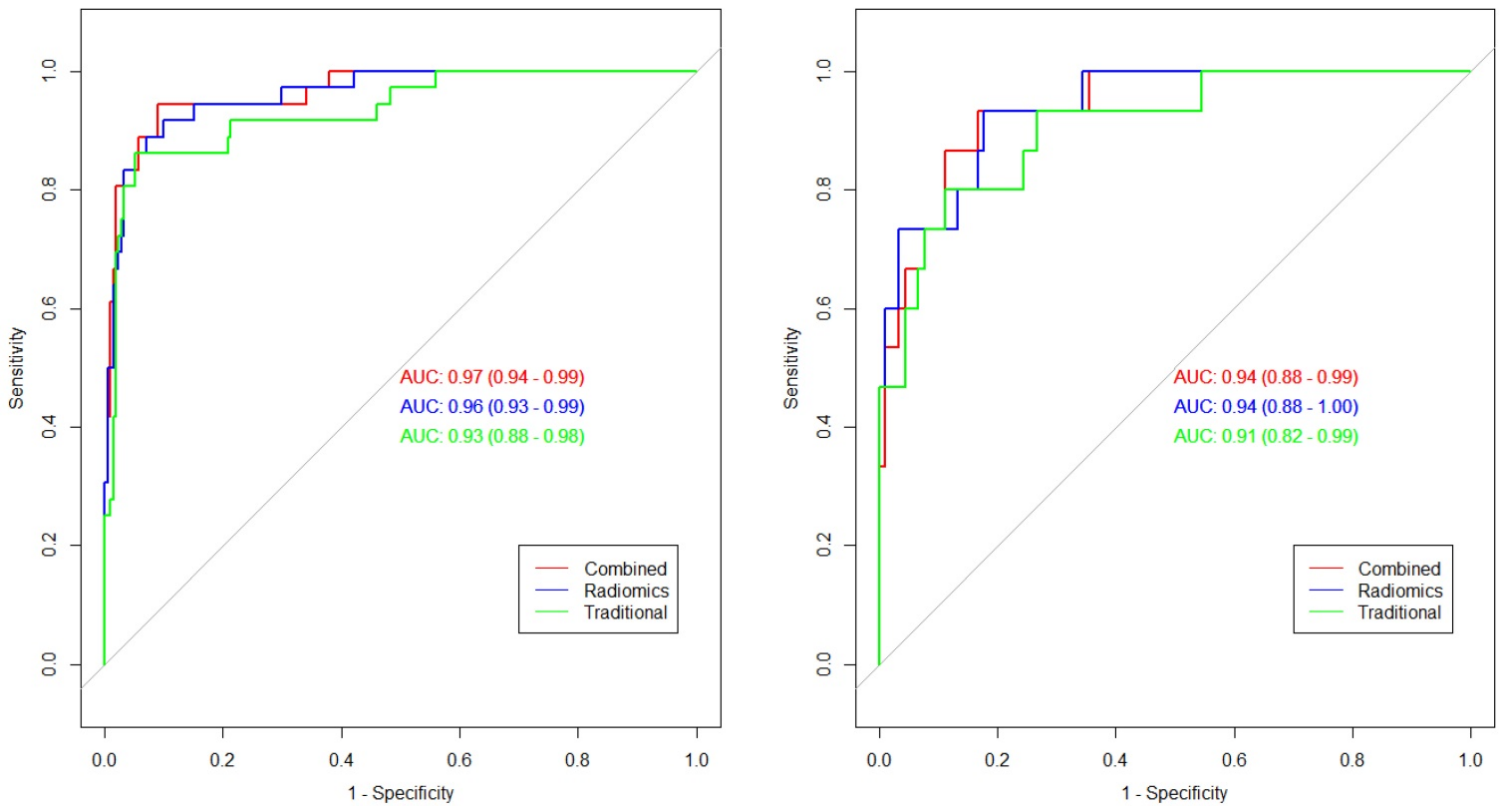
** Roc curves of the constructed models.** Green line represents the traditional quantitative model. Blue line represents the radiomics model. Red line represents the combined model.

Age, total volume, area proportion of Zone10 and radiomics score were selected to construct the combined model. The AUC in training set of quantitation model, radiomics model and combined model was 0.93, 0.96 and 0.97 respectively, while the AUC of the above three models in validation sets was 0.91, 0.94 and 0.94, respectively **(Figure 6)**. The radiomics model had the highest accuracy (92.31% in training sets and 83.81% in validation sets) and sensitivity (92.89% in training sets and 82.33% validation sets), while the combined model had the highest specificity (98.97% in training sets and 98.68% in validation sets). DeLong's test showed there was no difference in AUC between every two of ROC. More information on each model was demonstrated in **Table 3.**

**Figure 7:**
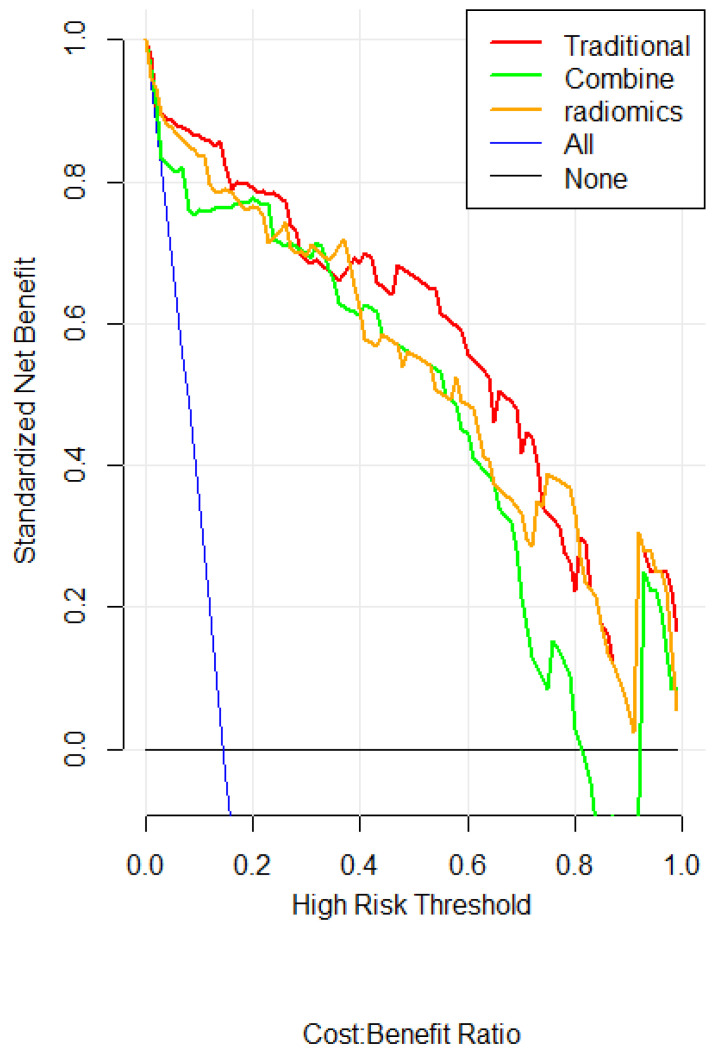
** DCA for the radiomics nomogram.** The y-axis represents the net benefit. The red, green and orange line represents the traditional, radiomics and combined model, respectively. The blue line represents the hypothesis that all patients had ARDS. The black line represents the hypothesis that no patients had ARDS. The x-axis represents the threshold probability. The threshold probability is where the expected benefit of treatment is equal to the expected benefit of avoiding treatment. For example, if the possibility of ARDS existence of a patient is over the threshold probability, then a treatment strategy for ARDS should be adopted.

The DCA curve showed that when threshold probability for a doctor or patients is within a range of 0 to 0.83, the combined model adds more net benefit than “treat all” or “treat none” strategies, while the traditional quantitation and radiomics model could add benefit in all threshold probability **(Figure 7)**.

## Discussion

ARDS existence is the major reason for ventilation care in COVID-19 patients. Besides, the current experience showed that earlier treatment of ARDS is one of the key measures to decrease the modality 9. Thus, early identification of ARDS existence could be beneficial to the COVID-19 patients. In this study, we constructed 3 different models by using the quantitative, radiomics and combined data. To the best of our knowledge, it was the first study to use the traditional quantitative and radiomics metrics to monitor the ARDS existence in COVID-19. Our results showed that used radiomics or quantitative metrics to monitor the ARDS existence was feasible, which had expanded the effectiveness of CT scans during the COVID-19 treatment, although it is still in controversy for reasons of availability, cost, and increased risk of cross-infection and radiation dosage 23.

There had already been some constructed model based on deep-learning to predict the prognosis of COVID-19 patient 24. Currently, all of the above models were based on the initial CT of the patient. Usually, this strategy did not cause significant bias because all patients were accepted similar treatment following the treatment guideline. However, as for COVID-19 patients, the treatment varies in different countries, different regions, even in the different patients of the same hospital because some drugs were proven to be ineffective after initial application. In this condition, the treatment strategy would cause significant heterogeneity. Thus, we used individual CT results during the patients' treatment instead of the initial CT to construct the model.

Our results showed that radiomics or traditional quantitative post-analysis on a CT image could add extra information of disease condition in COVID-19 patients. The traditional quantitation and radiomics data of chest CT had the potential to become a noninvasive method for ARDS screening. The DCA curves showed that radiomic or traditional quantitative model could add benefit to patients whatever the threshold probability, which means the model is better than the “treat all” or “treat none” strategies definitely. The noninvasive ARDS monitor method could benefit COVID-19 patients in many ways. Firstly, there are some patients with ARDS but without obvious respiratory symptoms, which was reported in some published studies^11^. Those patients could get earlier oxygen treatment and may have a better prognosis. Besides, the monitor method could also decrease the number of arterial punctures, which was an invasive procedure.

Although there was no difference in AUC among the three models, the radiomics model should be the most practical model for monitoring ARDS existence in COVID-19. Because for the traditional quantitation and combined model, the sensitivity was low (0.68 and 0.70, respectively), while the specificity was high (0.98 and 0.98, respectively). In contrast, the radiomics model had a relatively high sensitivity (0.94) and low specificity (0.86). Every model is not perfect, but sensitivity was much more important than specificity in ARDS monitoring because false-negative will cause delay of oxygen treatment to patients while false positive cause an unnecessary extra arterial puncture, which was much less harmful than the former.

Our traditional quantification result was homologous with the clinical findings. In our quantification result, the significant variable included: male (*P* =0.008), existence of hypertension (*P* = 0.016), total infection proportion (*P* <0.001), age (*P* =0.048) and area proportion of zone10 (CT value >100HU). Age and total infection proportion was the risk factor reported in many previous COVID-19 studies 10**.** The higher risk in male proportion might come from the higher smoking history of the male, which was reported as risk factor of bad prognosis of COVID-19 patients in previous study 25. The probable mechanism of higher ARDS rate in patient with hypertension was that after COVID-19 infection, the virus could combine with the ACE2 receptor, resulting in a decrease in the number of ACE2. Thus, when persons with hypertension get infected by COVID-19, their ACE2 receptor level will become extremely low since they have lower ACE2 receptor than those without hypertension 26, which could be a significant risk factor for lung failure 27. Finally, the infection regions with CT value >100 was highly related to the ARDS existence. The region with CT value >100 HU is seldom seen in pneumonia, it could refer to the dense fibrous tissue in the lung, which could be a sign of lung failure 28.

There were also several limitations in our study. The results could be influenced by the cohort retrospective nature. A larger sample of external validation is needed to acquire high-level evidence before clinical application. Besides, the cost-effective between radiation dose, medical cost and patients benefit should be analyzed further. Also, we had to mention that there is no “one fits” all analysis approach as performance of various ML workflows has been shown to depend on application and/or type of data. Thus, current study may change and improve by using different machine learning algorithms.

## Conclusion

A noninvasive ARDS existence monitoring model was constructed by using quantitative and radiomics analysis of chest CT images for COVDI-19 patients. Experimental results showed that the radiomics model was the most promising model for ARDS monitoring. Multi-center validation with a large number of samples is recommended in the future work.

## Supplementary Material

Supplementary figures and tables.

## Figures and Tables

**Table 1:** Characterize of the enrolled patients

Variables	Value
General characterize	
Total numbers	84
Age (year)	49.00 (34.00 -60.75)
Male	61.90%
Median time from illness onset to hospital admission (day)	5 (3 - 9)
Median time from admission to Frist CT (day)	1 (1 - 6)
Numbers of CT	4 (3 - 5)
ICU admission	13
Death	4
Initial symptom	
Fever	68
Cough	59
Sputum	16
Pharynagalgia	1
fatigue	3
headache	1
Dyspnoea	11
myalgia	5
stomachache	1
Diarrheal	2
Comorbidity	
COPD	6
Hypertension	20
Hyperlipemia	9
Cerebral infarction	4
Coronary heart disease	6
Cardiac dysfunction III-IV	7
Liver dysfunction	3
Diabetes	12
Chronic Kidney disease	5

**Table 2:** Summary of the quantitative metrics of enrolled CT image

Variables	No ARDS (N=51)		ARDS existence (N=301)		Total (N=352)	
	Mean	Sd	Mean	Sd	Mean	Sd
***Total Volume (%)	4.17	6.08	19.39	10.36	6.38	8.69
***Mean Density (HU)	607.59	121.71	-479.02	152.95	-588.96	134.34
***Zone1 Proportion (%)	0.56	1.00	1.40	1.33	0.68	1.10
***Zone2 Proportion (%)	0.30	0.47	0.84	0.70	0.38	0.54
***Zone3 Proportion (%)	0.21	0.33	0.70	0.51	0.28	0.40
***Zone4 Proportion (%)	0.14	0.24	0.54	0.37	0.20	0.30
***Zone5 Proportion (%)	0.10	0.18	0.44	0.30	0.15	0.23
***Zone6 Proportion (%)	0.07	0.14	0.37	0.27	0.12	0.19
***Zone7 Proportion (%)	0.06	0.11	0.34	0.28	0.10	0.18
***Zone8 Proportion (%)	0.05	0.10	0.34	0.29	0.09	0.18
***Zone9 Proportion (%)	0.06	0.15	0.50	0.48	0.13	0.27
***Zone10 Proportion (%)	0.03	0.07	0.26	0.28	0.06	0.15
***Vi Proportion (%)	2.18	2.97	8.41	5.09	3.08	4.01
***Vp Proportion (%)	2.00	3.37	10.97	7.95	3.30	5.36

Zonex indicated the volume proportion of the regions with specific density within the infection, including (zone 1: <-750 HU), (zone 2: from -750 HU to -650 HU), (zone 3: -649 HU to -550 HU), (zone 4: -549 HU to -450 HU), (zone 5: -449 HU to -350 HU), (zone 6: -349 HU to -250 HU), (zone 7: -249 HU to -150 HU), (zone 8: -149 HU to -50 HU), (zone 9: -49 HU to 100 HU) and (zone10:>100 HU). Vi represents the volume proportion of the infection located in the inner part of the lung, including anterior segment of bilateral upper lobes / apical segment of right upper lobe / lingular segment of left upper lobe / middle lobe of right lung/ anterior and medial basal segment of bilateral lower lobes. Vp represents the volume proportion of the infection located in the peripheral part of the lung, including posterior segment of right upper lobe / apicoposterior segment of left upper lobe / superior segment of bilateral lower lobes / lateral and posterior basal segment of bilateral lower lobes. *, **, ***, **** means the P value of t-test between two group >0.05, <0.05, <0.01, <0.001, <0.0001 respectively.

**Table 3:** Summary of the performance of each constructed model

Datasets	Accuracy	Sensitivity	Specificity
Traditional Train	93.52%	73.81%	97.56%
Traditional Test	87.62%	55.00%	95.29%
Radiomics Trian	92.31%	92.89%	88.89%
Radiomics Test	83.81%	82.22%	86.33%
Combined Train	91.50%	64.15%	98.97%
Combined Test	84.76%	58.28%	98.68%

## Abbreviations

CT: Computed tomography
COVID-19: Coronavirus disease 2019
SARS-Cov: Severe acute respiratory syndrome coronavirus
ROIs: segmented regions of interest
ARDS: Acute respiratory distress syndrome
ROC: Receiver operating characteristic
DCA: Decision curve analysis
AUC: The areas under the ROC curve
HU: Hounsfiled Unit
