# Supplementary material for: A Quantitative and Radiomics approach to monitoring ARDS in COVID-19 patients based on chest CT: a retrospective cohort study
Source: Int J Med Sci. 2020 Jul 6;17(12):1773–82. doi: 10.7150/ijms.48432 (PMC7378656; doi:10.7150/ijms.48432)
Supplement: Supplementary file 1 — Supplementary figures and tables. [file ijmsv17p1773s1.pdf]

Table S1: Parameters of the CT equipment

	GE Healthcare CT	Siemens CT
Kilovoltage	120 Kv	120 Kv
Slice thickness	0.625 mm	0.6 mm
Reconstruction	1mm	1mm
Rotation time	0.5s	0.5s
Inspiration breath hold	Yes	Yes
tube current	200~500mAs	110mAs

Table S2: Information of segmentation algorithm

	Training sets(N=247)	Validation sets (N =105)	P value
Age	51.35 ± 16.62	49.21 ± 17.042	0.274
Total infection volume proportion	6.37 ± 8.72	6.39 ± 8.67	0.980
Male proportion	139/247	49/105	0.107
ARDS existence	36/247	15/105	1.000

Table S3: Summary of the baseline of the training sets and validation sets

Accuracy Metrics	Mean	Standard deviation	Median	25% IQR	75% IQR	Number of infected samples
Dice Similarity Coefficient	91.6%	10.0%	92.2%	89.0%	94.6%	300
Volume Estimation Error (cm^3)	10.7	16.7	5.9	1.8	13.9	300
POI (The whole lung)	0.3%	0.4%	0.1%	0.0%	0.4%	300
POI (Left upper lobe)	0.4%	1.0%	0.1%	0.0%	0.4%	233
POI (Left lower lobe)	0.7%	1.6%	0.3%	0.1%	1.0%	267
POI (Right upper lobe)	0.3%	0.7%	0.1%	0.0%	0.5%	213
POI (Right middle lobe)	0.3%	0.7%	0.1%	0.0%	0.5%	204
POI (Right lower lobe)	0.6%	1.1%	0.3%	0.1%	0.9%	275
POI (Left upper lobe / posterior tip)	0.5%	1.0%	0.1%	0.0%	0.5%	189
POI (Left upper lobe / anterior)	0.5%	1.2%	0.2%	0.0%	0.5%	158
POI (Left upper lobe / upper tongue)	0.7%	1.7%	0.2%	0.0%	0.9%	192
POI (Left upper lobe / lower tongue)	0.7%	1.8%	0.2%	0.0%	0.8%	175
POI (Left lower lobe / dorsal)	0.9%	2.1%	0.4%	0.1%	1.2%	224
POI (Left lower lobe / anterior medial basal)	0.6%	1.4%	0.2%	0.0%	0.8%	209
POI (Left lower lobe / outer basal)	1.1%	2.5%	0.5%	0.1%	1.7%	228
POI (Left lower lobe / posterior basal)	1.1%	2.4%	0.5%	0.1%	1.6%	233
POI (Right upper lobe / apical)	0.4%	1.1%	0.1%	0.0%	0.5%	142
POI (Right upper lobe / back)	0.7%	1.7%	0.2%	0.0%	0.8%	186
POI (Right upper lobe /anterior)	0.4%	1.1%	0.1%	0.0%	0.9%	151
POI (Right middle lobe / lateral)	0.6%	1.5%	0.1%	0.0%	0.6%	183
POI (Right middle lobe / medial)	0.3%	0.8%	0.1%	0.0%	0.4%	167
POI (Right lower lobe / dorsal)	0.9%	1.9%	0.4%	0.1%	1.4%	233
POI (Right lower lobe / inner basal)	0.6%	1.4%	0.3%	0.1%	0.9%	162
POI (Right lower lobe / anterior basal)	0.6%	1.4%	0.1%	0.0%	0.9%	210
POI (Right lower lobe / outer basal)	0.9%	1.8%	0.4%	0.1%	1.2%	236
POI (Right lower lobe / posterior basal)	1.0%	2.0%	0.5%	0.1%	1.6%	249

Radscore：

0.647*original_firstorder_Maximum+-1.286*original_shape_MeshVolume+-4.531*original_shape_SurfaceVolumeRatio+-0.22*original_ngtdm_Complexity+-0.498*original_ngtdm_Busyness+1.281*original_glrlm_RunLengthNonUniformity+-2.12*original_firstorder_Skewness+-2.848*original_shape_Sphericity+-0.605*original_firstorder_TotalEnergy+1.224*original_gldm_DependenceNonUniformity+-1.327*original_gldm_DependenceEntropy+-0.571*original_shape_LeastAxisLength+0.177*original_firstorder_Minimum+-0.266*original_glszm_ZoneEntropy+-0.807*original_firstorder_InterquartileRange+-0.348*original_shape_MajorAxisLength+-0.691*original_firstorder_Kurtosis + -5.084
